# Repeated Femtosecond Laser-Assisted Astigmatic Keratotomies in Post-Keratoplasty Eyes

**DOI:** 10.3390/jcm11144221

**Published:** 2022-07-20

**Authors:** Nadav Levinger, Shmuel Levinger, Nir Erdinest, Asaf Achiron, Naomi London, Omer Trivizki, Eliya Levinger, Irina S. Barequet

**Affiliations:** 1 Hadassah Medical Center, Jerusalem 9574409, Israel; nir.erdinest@mail.huji.ac.il; 2 Enaim Medical Center Jerusalem, Tel Aviv 9438307, Israel; dr.s.levinger@enaim.co.il (S.L.); achironasaf@gmail.com (A.A.); eliya.levinger@gmail.com (E.L.); ibarequet@yahoo.com (I.S.B.); 3 Department of Ophthalmology, Tel-Aviv Sourasky Medical Center, Sackler Faculty of Medicine, Tel Aviv University, Tel Aviv 6997801, Israel; 4 Private Practice, Jerusalem 9422805, Israel; imnl4u@gmail.com; 5 Tel Aviv Sourasky Medical Center, Tel Aviv 6423906, Israel; omertr@gmail.com; 6 Goldschleger Eye Institute, Sheba Medical Center, Sackler Faculty of Medicine, Tel Aviv University, Tel Hashomer 5262000, Israel

**Keywords:** residual astigmatism, post-keratoplasty, femtosecond laser-assisted keratotomy, repeat astigmatic keratotomy

## Abstract

Visual rehabilitation after a keratoplasty is often compromised due to high amounts of residual astigmatism. Femtosecond laser-assisted astigmatic keratotomy (FLAK) is gaining popularity in decreasing this astigmatism. Though one set of two incisions suffices in most cases, sometimes the treatment proves insufficient and additional treatment is required. This case series evaluates the outcomes in patients who underwent two consecutive FLAK sets to correct post-keratoplasty residual astigmatism. All repeated FLAK cases performed on post-keratoplasty eyes were reviewed in a high-volume clinic as a non-comparative retrospective case series. The data extracted include the procedure parameters, time between procedures, refraction including sphere, astigmatism, spherical equivalent (SE), and uncorrected and best-spectacle-corrected distance visual acuity (UDVA, BSDVA, respectively). Eleven eyes of eleven patients aged 25.5 ± 10 treated with more than one FLAK procedure were identified. The average follow-up time was 6 months after the first FLAK and 24 ± 10 months after the second. The second FLAK was performed six months after the first. The preoperative mean astigmatism was −9.59 ± 2.36 D. At the final follow-up, the mean astigmatism decreased to −5.38 ± 1.79 D (*p* = 0.001). Repeated FLAK procedures achieved a significant and stable reduction of astigmatism in post-keratoplasty eyes. This procedure shows safety and effectivity in these complex eyes and may prevent the need for a further keratoplasty.

## 1. Introduction

Visual rehabilitation after a keratoplasty is often compromised due to residual astigmatism, often of a magnitude greater than 5 D [1,2]. This has been reported in up to 38% of post-penetrating keratoplasty (PKP) cases [3]. Possible treatment options are non-surgical (i.e., contact lenses and spectacles) or surgical [4,5,6,7]. One of the surgical options is known as arcuate keratotomy (AK). AK involves a series of incisions at approximately 80–90% corneal depth at the steep meridians of the mid-peripheral cornea [8,9,10,11,12]. This procedure can be performed manually or with a femtosecond (FS) laser (i.e., femtosecond laser-assisted astigmatic keratotomy or FLAK) [1]. For many years, AKs have only been performed by hand or by the Hanna keratome; however, the procedure lacks stability, has limited predictability, and, in rare instances, there have been associated intra- or post-operative complications [13,14,15,16,17,18,19]. 

The first group to describe the use of FS for AK was Nubile et al. [20], who showed that it is safe, effective, and reliable. Since then, multiple articles have shown similar results [21,22,23,24]. Although not yet an established consensus, many believe that using FS for AK is more reliable than manual AK performed with a Hanna trephine blade [23,25]. 

FLAK is known for its effectiveness and safety in decreasing corneal astigmatism after a penetrating keratoplasty (PKP) [9,18,26,27,28,29,30,31]. In many cases, one AK is usually effective in decreasing the amount of astigmatism. However, residual astigmatism after this procedure is common and may be significant, limiting maximal visual rehabilitation [8,26,27]. For patients who undergo a PKP or deep anterior lamellar keratoplasty (DALK), the primary goal of FSAK is to allow the patient to wear contact lenses or spectacles [7,9,32,33]. This study aims to investigate whether a second FLAK is a viable option to further reduce astigmatism after one procedure has proven inadequate. As far as the authors are aware, no other publication describes the outcomes of a second FLAK after a first FLAK to correct post-keratoplasty astigmatism.

## 2. Methods

### Ethical Principles

This study followed the tenets of the Helsinki Declaration. The approval of the institutional review board (IRB) of the Sheba Medical Center, Tel Hashomer, was obtained for this study and all procedures were carried out per their guidelines.

This is a non-comparative retrospective case series. All repeated FLAK cases performed in post-keratoplasty eyes by a single surgeon (SL) in a single clinic (Enaim Medical Center, Jerusalem, Israel) were reviewed.

## 3. Patients

A review of seven years of charts was performed, identifying 11 eyes of 11 keratoconus patients, 72% of which were male, who were treated with more than one FLAK procedure (Table 1). The data extracted included the procedure parameters, time between procedures, refraction including sphere, astigmatism, spherical equivalent (SE), and uncorrected and best-spectacle-corrected distance visual acuity (UDVA and BSDVA, respectively).

### 3.1. The FLAK Technique

All FLAKs were performed at the steep meridian with AMO’s IntraLase™ femtosecond laser (Johnson & Johnson, Santa Ana, CA, USA) at least six months after all sutures were removed, and graft–host junction stability was confirmed. The lengths of the cuts were 1/6 of the eye circumference on each side at a radius of 6 mm. The angle was 60 degrees and the depth was 80% of the corneal depth as determined by rotating Scheimpflug corneal tomography (Sirius, Costruzione Strumenti Oftalmici, Florence, Italy).

A beam energy of 3.2 to 3.4 J and a spot separation of 3 mm was utilized, lasting 5 s. Localin eye drops (Benoxinate HCl 0.4%, Dr. Fischer, Bnei Brak, Israel), a topical anesthetic, were applied a few minutes before the procedure.

The laser cutting procedure was performed after placing a suction ring on the patient’s eye, centered over the pupillary center. After completing the laser procedure, the incisions were widely opened via dissection of the remaining tissue bridges with a Sinskey hook.

The second AKs were performed six months after the first AK when best-corrected astigmatism and visual acuity proved inadequate. The same laser parameters were applied as the first AK, but the laser treatment was more central. The two cuts were performed at the steep meridian according to corneal topography. EyeSys (CAS, EyeSys Technologies, Houston, TX, USA) corneal topographic analysis was performed at 1, 3, 6, 12, and 24 months after the first AK and second AK. For this, EyeSys topography was utilized, as this was the imaging system that was available to monitor the patients at the assessment for the initial treatment.

### 3.2. Outcome Measurements

This study’s primary and secondary parameters were the resulting astigmatism and visual acuity. The data were abstracted at three time points, namely, at a baseline before the first FLAK (pre-AK 1), six months after the first FLAK and before the second FLAK (pre-AK 2), and at the last follow-up post-second AK (post-AK 2). At each of these points, the uncorrected distance visual acuity (UDVA), best-corrected visual acuity (BCVA), spherical equivalent (SE), and astigmatism were reviewed. Any reported complications were noted at each visit.

The astigmatism vectors were calculated with AstigMATIC software [34]. Analysis of the data was performed after the first FLAK (pre-AK 2) and after the second FLAK (post-AK 2). Overall vector analysis was performed following both FLAK procedures. In each analysis, the results were divided between low–medium astigmatism (0 to 6 D), medium–high astigmatism (6 to 10 D), and very high astigmatism (over 10 D).

## 4. Statistical Analysis

The statistical analyses of the pre-AK1, pre-AK2, and post-AK2 groups were performed using Statistical Package for Social Sciences 25.0 (SPSS Inc., Chicago, IL, USA) with two-tailed *t*-tests.

## 5. Results

### 5.1. Patient Characteristics

Eleven eyes of eleven patients treated with two FLAK procedures were identified, including eight male and three female patients. 

The indication for keratoplasty in 10/11 eyes was advanced keratoconus. One case underwent keratoplasty for a significant scar after penetrating trauma. Seven eyes had a penetrating keratoplasty (PKP) performed with the Hanna trephine (Moria Surgical, Antony, France). Two cases underwent PKP with a mushroom configuration using AMO’s IntraLase™ femtosecond laser (Johnson & Johnson, Santa Ana, CA, USA). Two cases had undergone a deep anterior lamellar keratoplasty (DALK) performed with AMO’s IntraLase™ femtosecond laser (Johnson & Johnson, Santa Ana, CA, USA).

### 5.2. Visual Outcome

The mean LogMAR UCVA for pre-AK 1 was 1.03 ± 0.29, and pre-AK 2 and post-AK 2 were 0.96 ± 0.31 and 1.00 ± 0.26, respectively. There were no statistically significant differences between UCVA pre-AK 1 vs. pre-AK 2 (*p* = 0.5905) or pre-AK 2 vs. post-AK 2 (*p* = 0.7464).

The efficacy index (post-operative UCVA/preoperative BCVA) values for post-AK 2 vs. pre-AK 1 and pre-AK 2 were 0.2 and 0.3, respectively. The safety index (post-operative BCVA/preoperative BCVA) values for post-AK 2 vs. pre-AK 1 and pre-AK 2 were 0.8 and 1.25, respectively.

LogMAR BCVA was 0.36 ± 0.25 (0.17–0.53) at pre-AK 1, 0.49 ± 0.31 (0.22–0.70) at pre-AK 2, and 0.41 ± 0.41 (0.12–0.53) at post-AK 2. There was no statistically significant difference between pre-AK 1 and pre-AK 2 (*p* = 0.2827), nor between pre-AK 2 and post-AK 2 (*p* = 0.6072). The mean sphere values at pre-AK 1, pre-AK 2, and post-AK 2 were 2.30 ± 2.19 D (0.00–7.00 D), 1.90 ± 2.78 D (9.00–21.00 D), and 1.70 ± 1.98 D (6.50–18.75 D), respectively (*p* = 0.55). The SE value increased with time, from a mean of 2.86 ± 1.44 D (0.50–5.00 D) at pre-AK 1 to a mean of 3.48 ± 2.04 D (0.50–7.38 D) at post-AK 2, (*p*= 0.37).

**Figure 1:**
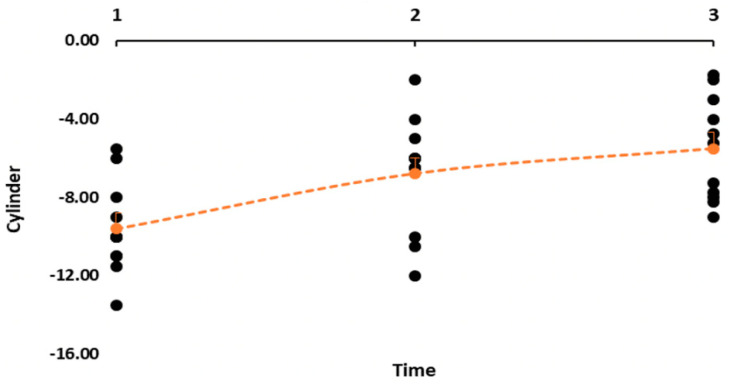
Comparison of the refractive cylinder between pre-AK 1 (1), pre-AK 2 (2) and post-AK 2 (3). Note the decrease of astigmatism between the three-time points, depicted by orange dotted line.

Evaluation of the astigmatism showed that at pre-AK 1, the mean astigmatism was −9.59 ± 2.36 D (−5.50–13.5 D). At pre-AK 2, it was −6.59 ± 1.99 D (−3.50–13.00 D). At post-AK 2, it was −5.38 ± 1.79 D (−1.75–9.00 D). The astigmatism pre-AK 2 was significantly lower than that at pre-AK 1 (*p* = 0.0043), and the astigmatism post-AK 2 was slightly but not statistically significantly lower than pre-AK 2 (*p* = 0.1494). When comparing the astigmatism for post-AK 2 to pre-AK 1, the post-AK 2 values are significantly lower (*p* = 0.001). (Table 1, Figure 1).

Table 2 summarizes the astigmatism and visual acuity outcome at the three time points, before the first Femtosecond laser-assisted astigmatic keratotomy (FLAK, Pre-AK 1), before the second FLAK (pre-AK 2) and after the 3rd FLAK (post-AK 2). The difference between the pre-AK 1 and post-AK 2 astigmatism was statistically significant (*p* = 0.001).

### 5.3. Vector Analysis

The vector analysis after the second FLAK showed the most accurate correction for low–medium astigmatism, followed by the high and very high astigmatism groups (Table 1). Vector analysis after the first FLAK procedure showed an overcorrection and a high angle error for eyes with low–medium astigmatism and more accurate outcomes for the higher levels of astigmatism.

**Figure 2:**
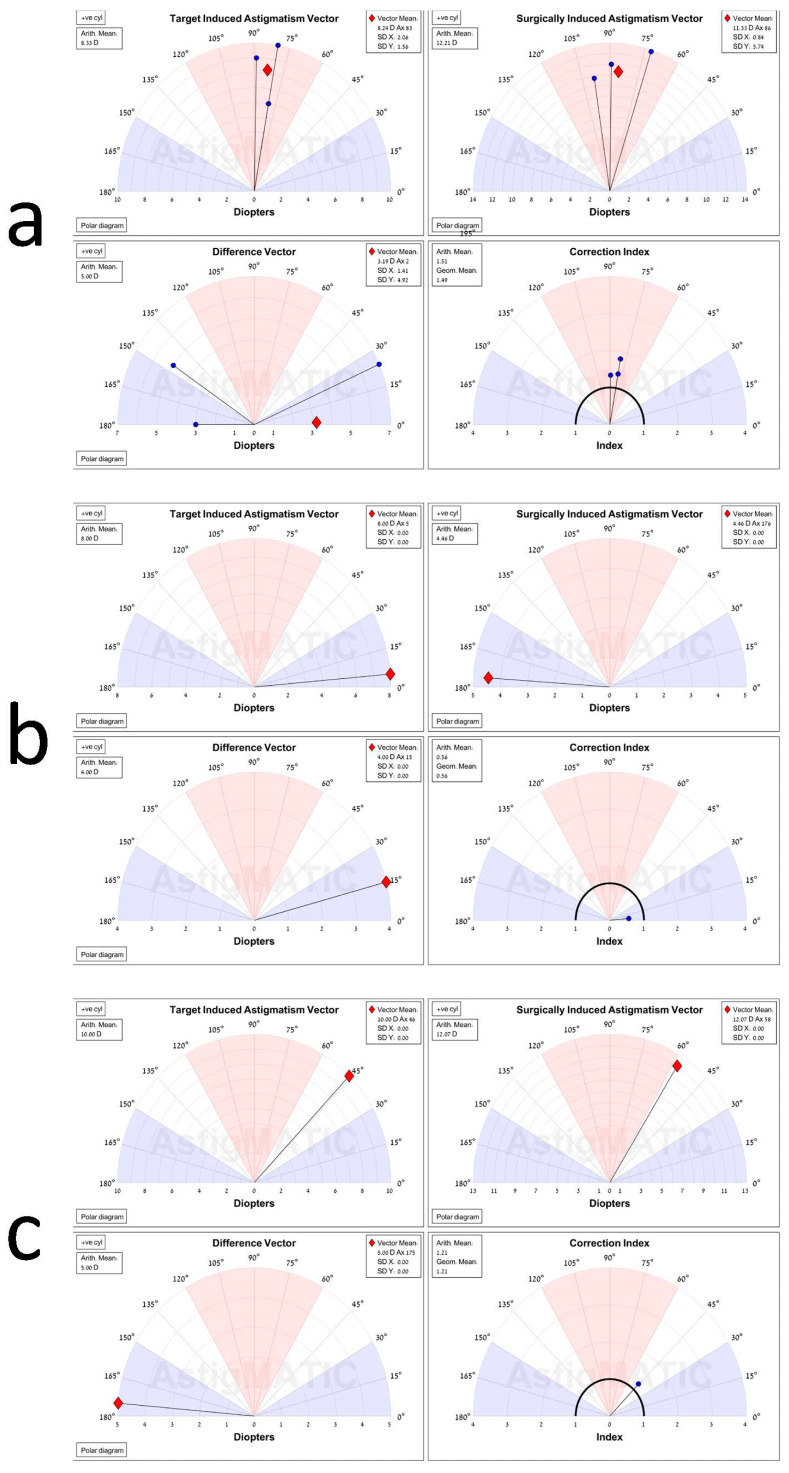
Printout results of the AstigMATIC software, comparing the residual astigmatism after the second astigmatic keratotomy (post-AK 2) to before the first keratotomy (pre-AK 1) on five patients: (**a**) Three patients exhibiting with-the-rule astigmatism. (**b**) One patient with against-the-rule astigmatism and (**c**) one patient with oblique astigmatism. The magnitude and axis of astigmatism are portrayed with a black line ending with a blue dot, or, in a graph depicting only one eye, with a red diamond, as it also represents the mean vector. The target-induced astigmatism (TIA) vector graph shows the range of astigmatism that the surgery intended to induce in this group at stage pre-AK 1. The surgically induced astigmatism (SIA) graph exhibits the range of achieved astigmatism, with both cylinder and axis treatment, which is post-AK 2 in this group. The difference vector (DV) graph portrays the residual astigmatism, summarizing the astigmatic error for both the magnitude and axis. This graph may be used as an absolute measure of the success of the treatment. The correction index (CI) graph shows the under or overcorrection of the astigmatism treatment. This can also measure procedure success and is calculated as SIA divided by TIA. When this value equals one, it represents an optimal surgical outcome. The value is greater or smaller than one if an overcorrection or undercorrection, respectively, occurs.

The AstigMATIC program does not include any eye with a surgically induced astigmatic (SIA) vector above 15 D. Therefore, data are not expressed for all patients here (Figure 2). In the three patients exhibiting with-the-rule astigmatism (Figure 2a), the difference between the target-induced astigmatism (TIA) as calculated pre-AK 1, and SIA as presented post-AK 2, was close, at 8.24 D and 11.33 D, respectively. The correction index (CI) was 1.51, close to 1, a value that would symbolize an optimal correction. The patient exhibited against-the-rule astigmatism (Figure 2b) and showed a difference between the SIA and TIA of 8.00 D and 4.46 D, respectively. The 95% CI here was 0.56, indicating a slight overcorrection. The patient with oblique astigmatism (Figure 2c) exhibited a low difference in magnitude of astigmatism between the TIA and SIA of 10.00 D and 12.07 D and a minimal disparity in the axis of 46 degrees in the TIA versus 58 degrees in the SIA. The CI was 1.21, indicating a very effective treatment outcome.

**Figure 3:**
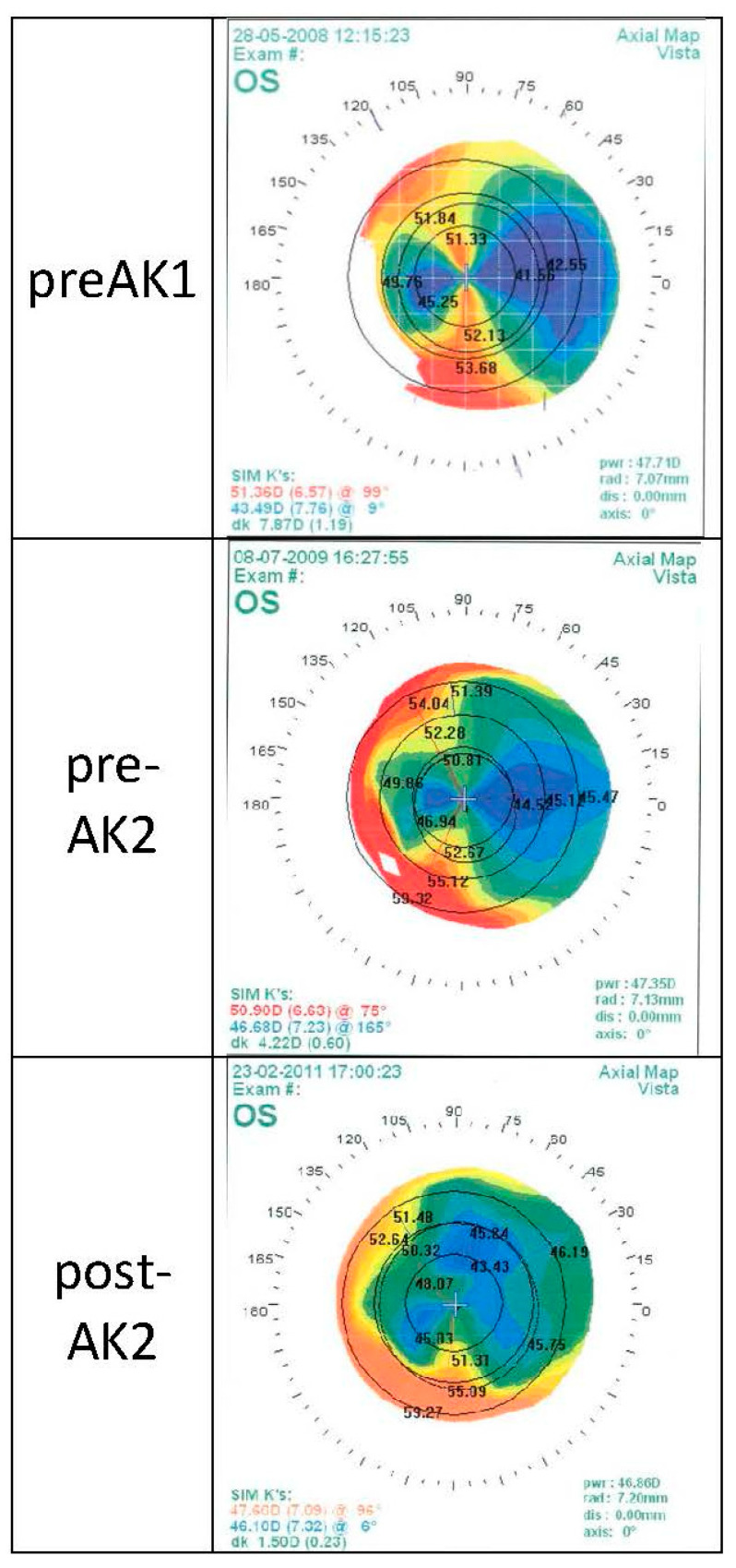
EyeSys topography maps of a patient that underwent repeated astigmatic keratotomy (AK) operations after a Penetrating keratoplasty (PKP). This patient had a positive outcome with a minor decrease in the astigmatism after the first AK treatment, and a significant decrease after the second AK treatment, and no significant change in the axis. Abbreviations: pre-AK1: before first AK procedure; pre-AK2: before second AK procedure; post-AK2: after second AK procedure.

EyeSys topography maps which were collected over three years for a patient who underwent repeated astigmatic keratotomy (AK) after PKP are presented in Figure 3. This patient benefitted from a favorable outcome, particularly after the second AK treatment, with a significant decrease in astigmatism after the second AK treatment and axis preservation.

## 6. Complications

There were no intraoperative or post-operative complications, including intra-/post-operative micro-perforation, macro-perforation, infection, or wound dehiscence in any of the procedures. Several patients had a change in the axis of astigmatism after treatment.

## 7. Discussion

This study has reviewed the outcomes of patients that have underwent two successive AK procedures using a femtosecond laser after a keratoplasty. A statistically significant reduction of astigmatism was achieved after the second FLAK.

Despite the high success rate in achieving anatomical clarity with corneal grafts [35], astigmatism remains a significant challenge for a satisfactory refractive outcome [28,36,37,38,39,40]. Multiple studies have demonstrated that residual astigmatism presents difficulties after treatments such as PKP, DALK, and handheld and FS-assisted keratoplasty [9,41,42,43,44,45].

Post-keratoplasty astigmatism is influenced by preoperative indication for surgery, the severity of the underlying disorder, intraoperative misalignment, healing of the graft host after suture removal, and intraoperative/post-operative complications [26].

In 2006, Poole and colleagues reported 50 eyes that underwent an AK with a diamond blade [46]. Although most of the procedures were resolved without complications, 8% of the patients developed complications (among them one micro-perforation, one graft host dehiscence), and 9 out 50 (18%) required a second procedure. The use of a FS laser for FLAK has been reported to have fewer complications and is usually safer than manual AK approaches [7,25,26].

The findings presented here for reduced astigmatism after the first FLAK (from a mean of 9.6 D to 6.78 D) and second FLAK (1.24 D), in addition to the overall decrease after the two successive FLAKS (4.06 D), support these previous findings. In this series, eleven eyes underwent a total of 44 cuts with no adverse events. Other studies that reviewed the FLAK procedure with only one arc set [47,48], where some have experienced complications (among them, one case of endophthalmitis and three corneal infections). However, in this study, the eyes underwent two sets of FLAK with no major complications [47,48]. This supports the hypothesis that corneal integrity can withstand four FLAK incisions with safe results.

The astigmatic magnitude decrease in the post-AK cornea is variable. It is influenced by the cut’s optical zone, length, and depth [49]. Most articles have concluded that approximately a 50% decrease can be expected after a paired AK, which is usually correlated with the pre-existing astigmatism [46,50]. Similar results have been exhibited with FLAK [15,48]. The average decrease after the first FLAK was 30% in this cohort. This lower magnitude is due to the more conservative parameters utilized in this study, such as a smaller arc size. The decrease in astigmatism was even lower (19%) after the second AK was performed. This is related to the smaller amount of residual astigmatism being treated and is in agreement with other previous reports [46,50,51].

This series has primarily been comprised of keratoconus patients with favorable results from both the first and second AK. Eyes treated after a traumatic injury had less favorable results, probably due to a highly irregular cornea involving the host region.

Many complex nomograms have been developed over the years to guide the architecture for an AK. In the current study, the nomogram was straightforward, as detailed above. A FLAK nomogram recently published by St Clair et al. considers different parameters and is more comprehensive [15]. The new nomogram may have great value and reduced outcome variability. This nomogram is based partly on FS laser parameters and, if it proves effective, will reinforce that FLAK is the superior alternative.

The shortcoming of FLAK primarily lies in its limited predictability, especially when trying to increase the arc size to achieve a more significant impact on an astigmatism [7,26,47,48]. Although this cohort has attained a smaller decrease in astigmatism after the first FLAK with a relatively small arc size, this more conservative approach to performing a second FLAK, if required later, helps prevent unwanted overcorrection. These results are similar to earlier studies that have used a smaller arc size and have had no resulting overcorrection [20,52]. Thus, using a smaller first arc and a second arc if needed to prevent overcorrection may prove a safer approach.

The long-term outcome of a FLAK or AK is uncertain. Fadlallah et al. [48] reported a change in astigmatism at a follow-up after FLAK, as did Böhringer and colleagues [53] after an AK. This cohort had a mean follow-up time of 24 months post-AK 2, a considerable amount of time, with satisfactory anatomical and refractive outcomes. The stability of the refractive outcome after 24 months may be due to the selection of two smaller arcs instead of one larger arc, which is possibly an additional reason to prefer this approach.

The FS laser’s safety and efficacy for an AK offers significant advantages for this semi-elective procedure. The alternative operations to this two-stage procedure, including two sets of FLAKs, is either excimer laser ablation, which offers less efficacy for correction of post-keratoplasty astigmatism [54], or a lens exchange with a toric intraocular lens [55]. The drawback of this approach is the risk of any intraocular surgical procedure, including possible corneal endothelial cell loss, the effect on accommodation, and may complicate a second PKP if needed in the future. In addition, an intrastromal corneal ring segment (ICRS) has been shown to be safe [56,57].

This study has some limitations. It is a retrospective case series of a relatively small cohort where eyes had undergone different types of keratoplasty, and not all procedures were performed by the same surgeon.

In conclusion, two consecutive FLAK procedures have shown in this case series to be safe, effective, and predictable surgical options for post-keratoplasty residual astigmatism, achieving an average astigmatism reduction of 4.06 D, with long-term stability and a low risk of overcorrection, ultimately allowing for improved optically prescribed outcomes. Furthermore, such treatment may prevent the need for an additional keratoplasty.

## Figures and Tables

**Table 1:** Patient data before keratoplasty, including their age, refraction, and graft diameter.

	Age	Spherical Refraction	Anterior Graft Diameter
AVG	25.5	1.5	8.107142857
SD	10.5	6.36	0.536523199
Min	15	−3	7.25
Max	51	6	8.6

Abbreviations: AVG: average; SD: standard deviation.

**Table 2:** Astigmatism and visual acuity values before and after the two Femtosecond laser-assisted astigmatic keratotomy (FLAK) procedures.

	Astigmatism		LogMAR UCVA		LogMAR BCVA	
	Mean (SD)	Range	Mean (SD)	Range	Mean (SD)	Range
**Pre-AK 1**	−9.59 (2.36)	−5.50 to −13.50	1.03 (0.29)	0.52–1.40	0.36 (0.25)	0.17–0.53
**Pre-AK 2**	−6.59 (1.99)	−2.00 to −13.00	0.96 (0.31)	0.30–1.30	0.49 (0.31)	0.22–0.70
***p*-value**	**Pre-AK 1 Vs. Pre-AK 2** 0.0043		**Pre-AK 1 Vs. Pre-AK 2** 0.5905		**Pre-AK 1 Vs. Pre-AK 2** 0.2827	
**Post-AK 2**	−5.38 (1.79)	−3.00 to −9.00	1.00 (0.26)	0.60–1.30	0.41 (0.41)	0.12–0.53
***p*-value**	**Pre-AK 2 Vs. Post-AK 2** 0.1494		**Pre-AK 2 Vs. Post-AK 2** 0.7464		**Pre-AK 2 Vs. Post-AK 2** 0.6072	

Abbreviations: SD: standard deviation; UCVA: uncorrected visual acuity; BCVA: best corrected visual acuity; Pre-AK1: before first AK procedure; Pre-AK2: before second AK procedure.

## Data Availability

Enaim Medical Center Jerusalem & Tel Aviv, 9438307, Israel.
